# Antibacterial Nanocomposite Ceramic Coating for Liquid Filtration Application

**DOI:** 10.3390/nano15120911

**Published:** 2025-06-12

**Authors:** Angelica Luceri, Michela Toppan, Alessandro Calogero, Antonio Rinaldi, Cristina Balagna

**Affiliations:** 1 Department of Applied Science and Technology, Politecnico di Torino, Corso Duca degli Abruzzi 24, 10129 Turin, Italy; cristina.balagna@polito.it; 2 CRAB Medicina Ambiente, Via Torino, 54, 13900 Biella, Italy; michela.toppan@outlook.it (M.T.); a.calogero@crab.it (A.C.); 3 TERIN Energy Technologies and Renewable Sources Department, ENEA (Agenzia Nazionale per le Nuove Tecnologie, l’Energia e lo Sviluppo Economico Sostenibile), Via Anguillarese, 301, 00123 Roma, Italy; antonio.rinaldi@enea.it

**Keywords:** composite coatings, water filters, antibacterial effect

## Abstract

Water contamination due to microbial proliferation remains a critical global challenge, especially with increasing urbanization, industrial activities, and the use of agrochemicals, and it requires the development of innovative methods for their purification that are not harmful to the environment and humans. In this study, innovative antibacterial nanocomposite coatings, composed of zirconia and silver nanocluster, were developed and deposited via eco-friendly co-sputtering physical vapor deposition (PVD) method onto electrospun polymeric membranes (PCL and PAN-PCL) for water filtration applications. Structural and morphological analyses, including XRD and UV-Vis spectroscopy, confirmed the deposition of a composite coating, consisting of an amorphous zirconia matrix embedding silver nanoclusters, homogeneously distributed on one side of the polymeric fibers. Wettability evaluations showed an increase in hydrophobicity after coating, particularly affecting the filtration performance of the PCL membranes. Antibacterial tests revealed strong inhibition against *Staphylococcus epidermidis* (Gram-positive) and partial efficacy against *Escherichia coli* (Gram-negative). Filtration tests of contaminated solutions revealed a 99% reduction in *Bacillus subtilis*, significant inhibition of *Listeria monocytogenes*, and limited effect on *E. coli*, with no bacterial proliferation observed on the coated membranes. These results underscore the effectiveness of ZrO_2_/Ag nanocomposites in enhancing microbial control and suggest a promising, scalable strategy for sustainable and safe water purification systems.

## 1. Introduction

The presence of pollutants in water represents a serious problem considering the toxicological effect that they could have on human health and on the environment. Access to clean and safe drinking water remains a critical challenge worldwide, particularly in developing regions, where over 2 billion people lack sufficient access according to UNICEF and WHO [1]. Increasing industrialization, urbanization, climate change, and rapid population growth intensify this issue, significantly impacting water availability in various regions, especially in Asia and Africa. The contamination of water sources leads to the transmission of numerous diseases, as improperly purified water often carries pathogenic microorganisms that pose serious health risks [2,3].

Microbial contamination occurs due to various sources, including surface water, wastewater, groundwater, and even household filtration systems. Studies have demonstrated the presence of bacteria such as *Streptococcus faecalis* and *Escherichia coli* in drinking water obtained from household refrigerators and cartridge filters [4,5]. Additionally, water infrastructure, including pipes, fittings, and faucets, can act as reservoirs for microbial growth, further compromising water quality [6]. Pollutants from soil infiltration and agricultural runoff introduce further microbiological and chemical contaminants, increasing the risk of waterborne diseases [7,8]. Conventional water purification methods, such as chemical disinfection with chlorine and ozone, present significant limitations, including the formation of potentially carcinogenic by-products and difficulties in recycling and reuse [9]. Alternative disinfection strategies, such as UV irradiation, also have drawbacks, including high energy consumption and the generation of harmful by-products [10]. Consequently, novel approaches that ensure effective microbial inactivation while minimizing environmental and health risks are urgently needed. Unlike traditional water purification methods that rely on chemical disinfectants, antibacterial nanocomposite coatings can provide long-term antimicrobial activity while reducing environmental impact and health risks. In order to avoid issues for human safety and the environment, green technologies could be evaluated, such as the PVD sputtering technique, which is a scalable, eco-friendly, and solvent-free process that ensures the uniform deposition of functional and high-purity coatings on several materials [11,12].

In this scenario, antibacterial coatings and nanomaterials could represent promising solutions to replace traditional water purification systems [13]. Among these, silver nanoparticles (AgNPs) play an important role, since their well-known and strong effect against a wide range of bacteria, viruses, and microorganisms [14,15,16,17] allows them to be involved in various applications [18,19,20,21]. Recently, metallic coatings, such as silver, copper, or aluminum, deposited via DC magnetron sputtering onto filter surfaces, have exhibited excellent adhesion and bactericidal properties while maintaining filter flexibility. The bacterial inactivation of *E. coli* and coliforms was moderate with zirconium and aluminum layers but significantly stronger with silver and copper coatings [22]. In particular, the role of silver nanoparticles in this field has been extensively studied due to their well-known great efficacy against bacteria and microorganisms. AgNPs deposited on membranes of polypropylene and poly(ether sulfone) (PES) demonstrate significant antibacterial activity against *E. coli*, with long-term efficacy persisting even after prolonged water exposure [23]. Additionally, silver nanoparticle-coated ceramic filters show a strong correlation between AgNP concentration and bacterial inhibition, particularly against Gram-positive strains. Flow tests confirmed that silver-modified filters achieve near-complete bacterial removal, preventing biofilm formation and ensuring sustained disinfection [24]. Alternative polymeric coatings, such as sulfonated pentablock copolymers (s-PBC), exhibit a pH-dependent bactericidal effect against Pseudomonas aeruginosa, enhancing filtration efficiency through surface charge modulation [25]. Innovative water purification strategies also include microsphere-based filtration systems. Silver-coated poly(methyl methacrylate) (PMMA) microspheres exhibit strong antibacterial activity, achieving up to 94% bacterial reduction at low nanoparticle concentrations and complete bacterial elimination at higher concentrations. Notably, filtration systems composed of Ag-coated microspheres maintain their effectiveness over multiple uses, with minimal performance degradation after prolonged operation [26]. Recent studies have demonstrated the applicability of zirconia-based materials in water filtration systems owing to their excellent chemical and thermal stability, resistance to biofouling, and long-term durability in aqueous environments [24].

This study aimed to develop silver/zirconia nanocomposite coatings to be deposited on polymeric membranes for liquid filtration as efficient alternatives to conventional water purification methods. The developed nanocomposite coatings, obtained by optimizing the PVD co-sputtering process parameters, are made up of a zirconia matrix embedding silver nanoclusters to confer an antibacterial effect. The use of silver nanoclusters embedded in a ceramic zirconia matrix combines the well-known antimicrobial efficacy of silver with the chemical stability of zirconia, offering a durable and safe solution for water filtration. Morphological, compositional, and structural analyses confirmed the success of the deposition process. Antibacterial evaluations were performed to assess the coating’s effect against Gram-positive and Gram-negative bacterial strains. In addition, an experimental setup was developed to assess the effect of the silver/zirconia coatings in the filtration of aqueous solutions.

## 2. Materials and Methods

### 2.1. Coating Deposition

In this study, two electrospun polymeric membranes for liquid filtration were selected as substrates for the deposition of an antibacterial ceramic nanocomposite coating and provided by Nanofaber S.r.l. (Roma, Italy). The first membrane, hereafter referred to as “PCL”, was manufactured via solution electrospinning from a 12% wt polycaprolactone (PCL) (Nanofaber, Italy, MW = 80,000, medical grade) in dimethylformamide (DMF) (≥99.8% ACS, VWR Chemicals, Radnor, PA, USA), and was fabricated under relative humidity conditions of 27–28% using a LE100 electrospinning station (Fluidnatek, Valencia, Spain) equipped with an environmental control chamber. The second membrane, hereafter referred to as “PAN-PCL”, consisted of a blend of 70% polyacrylonitrile (PAN) (powder, Merck, Darmstadt, Germany, MW = 150,000) and 30% of said PCL, and was manufactured from a 12% wt DMF solution by electrospinning under relative humidity conditions of 35%. Both the membranes had a thickness ranging from 40 to 140 µm.

The deposition of the zirconia/silver nanocluster composite coatings followed a patented process [27] based on a co-sputtering technique. The sputtering system was equipped with two targets working simultaneously: one composed of zirconia for the coating matrix (99.98% ZrO_2_, Nanovision^TM^, Beijing, China) and the other one of silver (Franco Corradi S.r.l., Rho, Italy, 99.9% purity). A radio frequency (RF) power of 250 W was applied to the zirconia target, while a direct current (DC) power of 4 W was applied to the silver target to obtain silver nanoclusters. The coating deposition process occurred in a pure argon atmosphere at a pressure of 5.5 dPa, and lasted 10 and 20 min. In the text, the two coatings will be named ZrO2_Ag_10 and ZrO2_Ag_20, referring to the different times of deposition.

### 2.2. Chemical, Morphological, and Structural Coating Characterization

An Energy-Dispersive Spectroscopy (EDS, EDAX PV 9900^TM^, Mahwah, NJ, USA) analysis was performed to determine the composition of the coatings, specifically the atomic percentages of Zr and Ag, which constitute the matrix and nanoclusters of the coatings. Three distinct areas were examined at a voltage of 15 kV and at a magnification of 150× to obtain a statistically representative quantification of the elements.

Morphology was observed via field emission scanning electron microscopy at different magnifications, using a LEO1530 (Zeiss, Jena, Germany) for the pristine membranes and QUANTA INSPECT 200 (Zeiss SUPRA 40, Jena, Germany) on the coated membranes. Differences in membrane aspect before and after the coating depositions were detected.

Structural evaluations were conducted using X-ray diffraction (XRD) with a Bragg–Brentano X’pert Philips diffractometer on coatings deposited onto a soda-lime glass substrate (used as model substrate). The analysis was performed in the X-ray grazing incidence mode, maintaining a constant low angle of 1°, in order to determine the crystalline phases of the matrix and metallic nanoclusters within the coatings This approach allowed for the selective detection of information from the coating alone, minimizing interference from the substrate.

Additionally, a UV–Visible absorption analysis (UV–Vis, Shimadzu UV-2600, Kyoto, Japan) was performed on the coated soda-lime glass to investigate the presence of silver nanoclusters. The analysis was conducted over a wavelength range of 200–700 nm.

### 2.3. Contact Angle

The wettability of the uncoated and coated membrane surfaces was evaluated through contact angle measurements using the sessile drop method. A drop of water was placed on the surfaces of the uncoated PAN and PAN-PCL membranes, as well as on the ZrO2_Ag_10 and ZrO2_Ag_20 coatings, using a syringe. The contact angle was calculated by capturing and analyzing the images with the Image J software 1.54k. For each sample, the measurement was performed in triplicate, and the related media and standard deviation were calculated.

### 2.4. Antibacterial Evaluations

The antibacterial effect of the zirconia-based coatings with silver nanoclusters was qualitatively assessed using the inhibition halo test following the NCCLS M2-A9 performance standard [28]. *Staphylococcus epidermidis* (Gram-positive, ATCC 14990) and *Escherichia coli* (Gram-negative, ATCC 8739) colonies were inoculated in Mueller–Hinton broth to obtain a bacterial suspension with a McFarland turbidity standard of 0.5 (1 × 10^8^ CFU/mL), which was then uniformly spread onto Mueller–Hinton agar. Both the uncoated and coated samples were placed in direct contact with the agar and incubated at 35 °C for 24 h. The antibacterial activity was determined by the presence of a clear halo around the sample, indicating bacterial growth suppression.

### 2.5. Bacterial Filtration and Culture Test Procedure

An experimental setup was specifically designed to evaluate the bacterial filtration performance of membranes. Three bacterial strains, *Bacillus subtilis* subsp. *spizizenii* (0.4–0.5 µm, Gram-positive), *Listeria monocytogenes* (serotype 4b) (1 µm, Gram-positive), and *Escherichia coli* (1–2 µm, Gram-negative), were tested. *B. subtilis* and *E. coli* were obtained from MICROBANK^TM^ porous beads (Pro-Lab Diagnostics, Round Rock, TX, USA) previously inoculated and stored at −70 °C. The beads were immersed in 100 mL of sterile MRD, allowing bacterial release into the solution. *L. monocytogenes* was prepared by incubating a pre-contaminated red bead in MRD for 24 h, followed by a 1:100 dilution in distilled water.

The system was composed of a filtration funnel, a collection container housing a sterile Pyrex bottle, a vacuum pump (operating at 40 kPa), and a clamp to secure the membrane between the funnel and the container. All the procedures were performed under a laminar flow hood to ensure sterility.

The procedure consisted of filtering the bacterial solution using the described apparatus. In order to control the amount of bacteria present in the solution, an aliquot (100 µL) of both unfiltered and filtered bacterial solutions was inoculated and spread onto Tryptic Soy Agar (TSA, EP) plates. The agar was incubated at 30 °C for 24 h, and then the colony-forming units (CFUs), grown on the agar, were counted. The contaminated membranes were also put on the Tryptic Soy Agar in order to verify the potential bacterial growth on the surface of the substrates after 24 h of incubation at 30 °C. The Tryptic Soy Agar was prepared with casein peptone (15.0 g/L), soybean peptone (5.0 g/L), sodium chloride (5.0 g/L), and agar (15.0 g/L). The diluent used was Maximum Recovery Diluent (MRD, 3 × 3 L) containing peptone (1.0 g/L) and sodium chloride (8.5 g/L), according to the ISO 6887-1 standards [29] for the recovery of stressed microorganisms.

## 3. Results and Discussion

### 3.1. Chemical, Morphological, and Structural Coating Characterization

**Figure 1:**
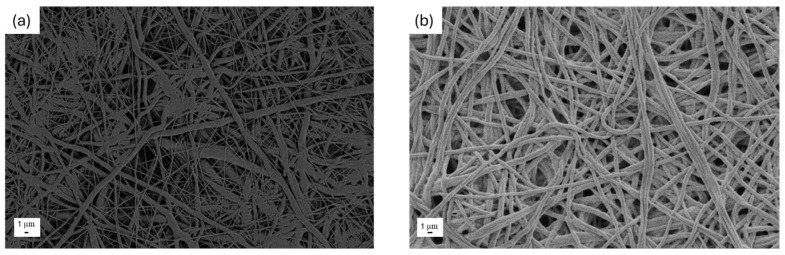
Morphological images at low magnification of uncoated (**a**) PCL and (**b**) PAN-PCL membranes.

In Figure 1, the morphology of the obtained electrospun PCL (Figure 1a) and PCL-PAN (Figure 1b) is shown before the coating deposition process. At low magnification, both membranes appear similar and macroscopically dense, consisting of a thick network of randomly oriented, intertwined fibers.

The zirconia-based coatings were deposited on one side of the membrane only, specifically, the side intended to be in contact with the liquid to be filtered. The opposite side was intentionally left uncoated. This approach was adopted not only to enable selective surface functionalization but also to preserve the integrity of the polymeric membrane, which could otherwise be compromised due to thermal sensitivity or mechanical stress during the deposition process.

The deposition of the silver/zirconia nanocomposite coatings occurred successfully on the polymeric membranes, as confirmed by the EDS analysis. The amount of zirconium and silver in the ZrO2_Ag_10- and ZrO2_Ag_20-coated samples is shown in Table 1. The uncoated samples are mainly composed of C and O (quantitative compositions not reported).

**Figure 2:**
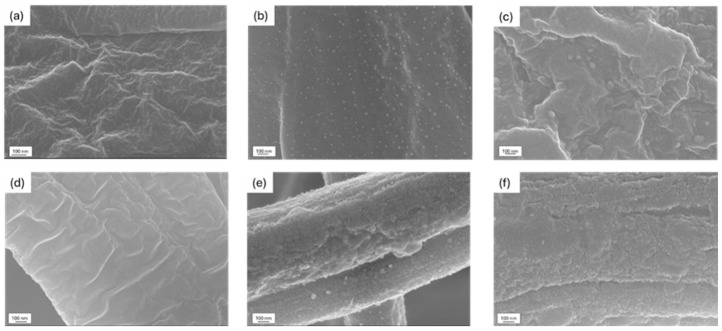
FESEM images of (**a**) uncoated PCL; (**b**) ZrO2_Ag_10 and (**c**) ZrO2_Ag_20 coatings on PCL membrane; (**d**) uncoated PAN-PCL; and (**e**) ZrO2_Ag_10 and (**f**) ZrO2_Ag_20 coatings on PAN-PCL membrane.

Increasing the deposition time led to a higher amount of Ag: the low standard deviation values suggest a homogeneous coating deposition, which is further confirmed by FESEM analysis (Figure 2). The PCL-coated membrane (Figure 2b,c) showed small particles, likely zirconia aggregates, dispersed homogeneously on a smooth surface, while the PCL-uncoated fibers (Figure 2a) appeared rough and lacked a uniform surface. A similar trend was observed for the PAN-PCL membrane, where the coating was clearly distinguishable and exhibited a globular structure (Figure 2e,f). For both cases, increasing the deposition time did not result in morphological differences, suggesting that the process yields a consistent and homogenous coating regardless of duration.

The presence of silver nanoclusters, which were not visible in FESEM images but were identified through the EDS analysis, was further confirmed by structural evaluations. The UV-Vis and XRD analyses were performed on the ZrO2_Ag_10 and ZrO2_Ag_20 coatings deposited on a soda-lime glass substrate, verifying the incorporation of silver nanoclusters within the zirconia matrix.

**Figure 3:**
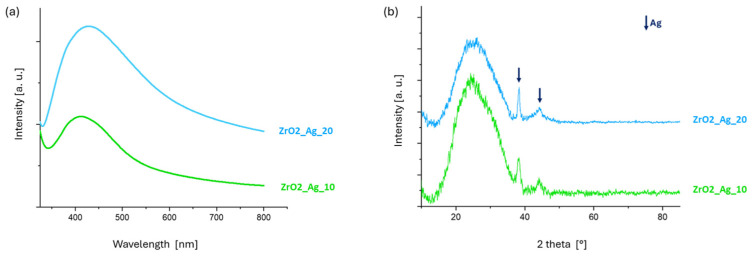
(**a**) UV-Vis and (**b**) XRD analyses of ZrO2_Ag_10 and ZrO2_Ag_20 coatings.

In the UV-Vis spectra (Figure 3a), a distinct absorption peak is observed in the range of 414–420 nm for both samples, characteristic of the surface plasmon resonance (SPR) of silver nanoclusters embedded within the zirconia matrix [30].

The XRD patterns reported in Figure 3b confirm the amorphous nature of the deposited zirconia matrix, as evidenced by the presence of a broad diffuse halo centered around 2θ ≈ 25°, which is typical of amorphous zirconium oxide [31]. In contrast, two distinct diffraction peaks are observed at 2θ values of approximately 38° and 44°, corresponding to metallic silver [32], according to the PCPDF 01-089-3722 code. The curves for 10 and 20 min deposition are very similar, and the intensity of these peaks remains comparable between the two coatings. This suggests a similar silver content and nanoparticle size, as both deposition times are relatively short. Consequently, any variations in the size of the silver nanoparticles are expected to be minimal. While pure zirconia films deposited by sputtering are generally crystalline, co-deposition with other materials has been reported to promote the formation of an amorphous structure, an effect that is likewise observed in the present study [31,33]. Additionally, XPS analyses from previous work confirmed the formation of zirconia during the deposition process [33]. Similar co-deposition strategies, involving silver nanoclusters embedded in either silica or zirconia matrices, have been successfully employed for the fabrication of functional coatings in air filtration systems [33,34,35].

In this study, zirconia was deliberately selected as the matrix material for the nanocomposite coatings due to its exceptional combination of mechanical robustness, chemical inertness [36], and outstanding stability in aqueous environments [33], as consistently demonstrated in prior work. These intrinsic properties render zirconia an ideal candidate for applications requiring long-term durability under harsh and humid conditions [37]. In addition to its structural advantages, zirconia plays a fundamental role in stabilizing and supporting silver nanoparticles, which are responsible for antibacterial activity. Specifically, the zirconia matrix prevents the agglomeration of the nanoparticles and protects them from rapid oxidation or deactivation, thereby enabling a gradual and sustained release of Ag⁺ ions over time [33]. This controlled ion release is crucial for maintaining long-term antibacterial effectiveness.

A previous study [33] reported a coating deposition process lasting approximately one hour, yielding coatings with a thickness of around 100 nm. In the present work, although the coating thickness was not directly measured, the significantly reduced deposition times of 10 and 20 min, necessitated by the high thinness and thermal sensitivity of the electrospun PCL and PAN_PCL membranes, suggest that the resulting coatings are expected to be thinner than those previously reported. Nonetheless, despite the shorter processing durations, the deposition still produced thin and homogeneous coatings containing silver nanoclusters, as evidenced by the characterization results, which are comparable to those obtained in prior studies with longer deposition times.

### 3.2. Contact Angle

**Figure 4:**
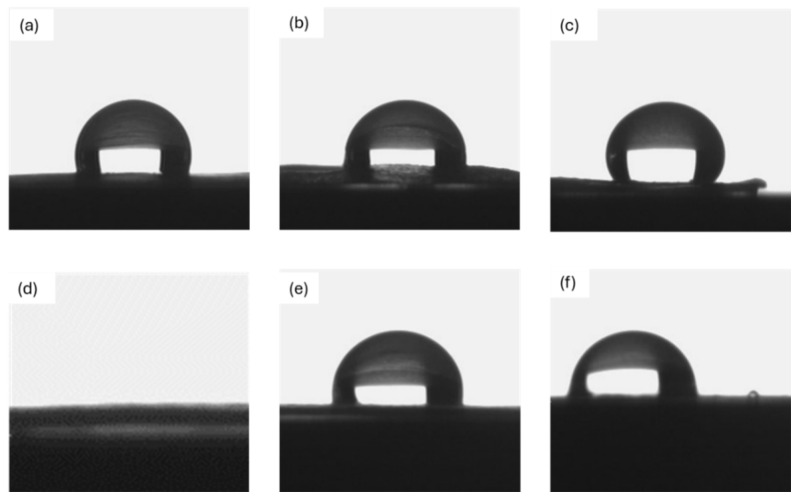
Contact angle with distilled water on (**a**) uncoated PCL; (**b**) ZrO2_Ag_10 and (**c**) ZrO2_Ag_20 on PCL; (**d**) uncoated PAN-PCL; and (**e**) ZrO2_Ag_10 and (**f**) ZrO2_Ag_20 on PAN-PCL.

Since the studied membranes are intended for prolonged use in contact with a liquid solution, the wettability of the uncoated and coated membranes was evaluated. The corresponding results are shown in Figure 4 and the values are listed in Table 2.

Uncoated PCL exhibited hydrophobic behavior toward water, as indicated by a contact angle of approximately 107°, consistent with the results reported in a previous study [38], where similarly fabricated pure fibrous PCL showed comparable wettability. For PCL samples with Ag nanoparticles deposited on the surface, higher contact angles of approximately 129° and 134° were observed. However, no evaluation was possible for the PCL-PAN membrane, as the water drop was immediately filtered thanks to its high hydrophilicity. In contrast, all the silver/zirconia nanocomposite coatings demonstrated high contact angle values, ranging from about 98° to 127°, which are similar to the values reported for RF-sputtered zirconia coatings by Chauhan et al. [39]. This suggests that the zirconia-based coating conferred hydrophobic behavior to the membranes, significantly reducing the filtration rate. While hydrophilic membranes may facilitate fluid penetration and increase flow rate, hydrophobic and superhydrophobic surfaces have been shown to reduce bacterial adhesion and biofilm formation, particularly when combined with intrinsic antibacterial agents such as silver nanoparticles [40]. Therefore, while the increase in contact angle may reduce the filtration rate in highly hydrophobic membranes such as PCL, it also contributes to the antibacterial performance by limiting microbial attachment, an important consideration for water purification systems.

### 3.3. Antibacterial Evaluations

**Figure 5:**
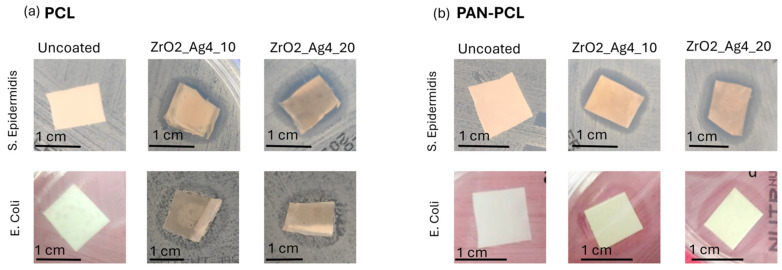
Inhibition halo test against *S. epidermidis* and *E. coli* on uncoated and ZrO2_Ag_10- and ZrO2_Ag_20-coated (**a**) PCL membrane and (**b**) PAN-PCL membrane.

The antibacterial effect of the silver/zirconia nanocomposite coatings was evaluated through an inhibition halo test performed on *S. epidermidis* and *E. coli*, and the results are shown in Figure 5. A comparison with the uncoated membranes is also reported.

Proliferated bacterial colonies are visible around both the uncoated PAN and PAN-PCL membranes, confirming the absence of intrinsic antibacterial properties. However, both the silver/zirconia nanocomposite coatings demonstrated effective antibacterial activity against *S. epidermidis*, as evidenced by the bacteria-free zone surrounding the coated samples. Despite ZrO2_Ag_10 being obtained with a shorter process, resulting in lower silver content compared to ZrO2_Ag_20, the bacterial inhibition of *S. epidermidis* was achieved with the formation of bacteria-free zones similar in size. Regarding the *E. coli* strain, a fully bacteria-free zone around coated membranes was not observed. However, bacterial colony proliferation was reduced and slowed down, indicating partial antibacterial activity. The observed reduction in bacterial colonies by the coated membranes suggested a strong antibacterial effect towards the Gram-positive strain, *S. epidermidis*, and a partial antibacterial activity against Gram-negative bacteria, consistent with previous research by Rodrígues et al. [41], who found that silver-doped zirconia filters exhibited efficacy against *S. aureus* but not against *E. coli*. In a previous study [33], the antibacterial activity of co-sputtered composite coatings with SiO_2_, ZrO_2_, or Al_2_O_3_ matrices containing different amounts of silver nanoclusters was compared, demonstrating the effective inhibition of *E. coli*. However, the effectiveness towards P. aeruginosa strongly depended on the amount of Ag in the coating. In the presented study, the deposition times were significantly reduced to avoid damaging the thermosensitive membranes during the process, and this also affected the amount of Ag nanoclusters inside the coating. As *E. coli* is considered a stronger bacterium, it can be assumed that the lower content of Ag nanoclusters is likely insufficient for the complete inhibition of *E. coli*, in contrast to what occurs with *S. epidermidis*. Capillary membranes composed of silver nanoparticle (AgNP)-doped zirconia have demonstrated excellent efficiency in filtering solutions contaminated with *E. coli*, showing effective bacterial retention and bactericidal activity against the retained microorganisms [42]. Moreover, silver nanoparticles incorporated into a hybrid nanocomposite framework derived from medical leaves have shown promising results in water purification, exhibiting strong antibacterial effects against both *S. aureus* and *E. coli* while also offering good fouling resistance [43]. The differences in the results against Gram-negative strains may likely be attributed to the specific system developed, to the amount of silver in the coatings, and to the mechanism involved in the antibacterial activity, since in previous work [33], the antibacterial effect of Ag NPS deposited via PVD technique on PCL substrate showed strong effect against *E. coli*.

### 3.4. Bacterial Filtration Efficiency

The bacterial filtration efficiency of PAN-PCL membranes with and without ZrO2-Ag coatings was evaluated using a solution containing *Bacillus subtilis*, *Listeria monocytogenes*, or *Escherichia coli*. This test was not fully performed on the PCL sample alone, as the filtration of the aqueous bacterial solution proved to be extremely difficult, likely due to its highly hydrophobic nature. Therefore, only the PAN-PCL sample was tested. The results of the number of colony-forming units (CFUs) counted from the solution before and after filtration and the proliferation on membranes are reported in Table 3.

**Figure 6:**
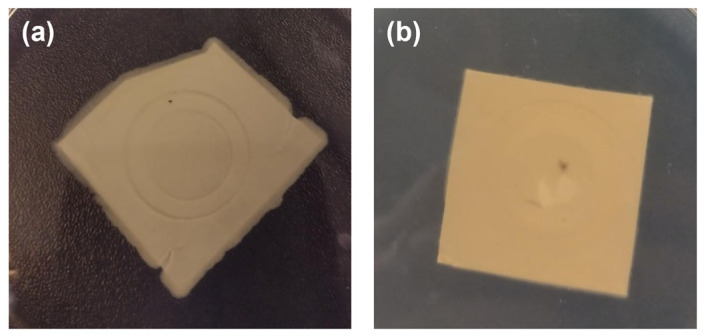
Photographs relative to the PAN-PCL membranes contaminated by *B. subtilis* after 24 h of incubation: (**a**) uncoated; (**b**) ZrO2_Ag_10 coated.

For *B. subtilis*, the PAN-PCL membrane demonstrated a remarkable 99% bacterial reduction, decreasing from 88 CFUs in the unfiltered solution to 1 CFU after filtration. However, a significant bacterial proliferation was observed on the membrane surface after 24 h of incubation (Figure 6a). Even if the CFUs relative to ZrO2_Ag_10 on the PAN-PCL membrane are invalidated due to an excessive dilution of the solution for the counting, the sample results show free bacteria after 24 h of incubation (Figure 6b).

For the PAN-PCL membrane with the ZrO2_Ag_20 coating, the difference in CFUs is evident before and after filtration, and no colonies were observed on the surface of the membrane.

In the case of *L. monocytogenes*, the PAN-PCL membrane showed extensive bacterial growth, which was difficult to count. The membrane side exposed to the bacterial solution showed substantial growth. In contrast, the coated membranes reduced CFUs from 27 in the unfiltered solution to 14 in the filtered one, with no bacterial proliferation on the membrane in both cases. For *E. coli*, the uncoated membrane reduced the CFUs during filtration; instead, the coated membranes showed limited filtration. However, both the uncoated and coated membranes demonstrated an effective antibacterial surface action, with no colony growth on the membrane itself. These results confirm that the deposited nanocomposite coatings have a stronger antibacterial effect against Gram-positive strains, with a quasi-complete reduction in *B. subtilis* colonies, and a partial reduction in *L. monocytogenes* colonies, while for *E. coli*, a complete antibacterial activity was not attained.

## 4. Conclusions

This study successfully developed and characterized zirconia-based nanocomposite coatings incorporating silver nanoclusters applied to polymeric membranes via co-sputtering PVD for water filtration purposes. Despite the reduced deposition time required to preserve the integrity of the thermosensitive membranes, the coatings showed a uniform distribution and effective antibacterial properties. The inhibition halo tests confirmed high efficacy against *S. epidermidis* and the partial inhibition of *E. coli*, while the filtration experiments demonstrated substantial bacterial reduction for *B. subtilis* and *L. monocytogenes*, with limited activity against *E. coli*. The results highlight the relevance of silver content and membrane hydrophobicity in achieving antibacterial effectiveness. Overall, these nanocomposite coatings represent a viable and eco-friendly alternative to conventional chemical disinfection techniques, especially for enhancing the safety of water filtration systems.

Future studies will explore the optimization of silver content and deposition parameters to improve the efficacy against Gram-negative strains such as *E. coli*. In addition, further research will focus on expanding the antibacterial evaluation to a broader range of pathogens, including antibiotic-resistant strains. Future efforts will be dedicated to scaling up the PVD co-sputtering process for industrial applications and evaluating its integration into the existing water purification systems. Finally, the environmental sustainability of the coatings will be studied, particularly focusing on the potential for membrane regeneration or recycling.

## Figures and Tables

**Table 1:** Compositional analysis of zirconia/silver composite coatings deposited on water filters for 10 and 20 min.

Substrate	Deposition Time	Zr (% at.)	Ag (% at.)
PCL	10 min	4.5 ± 0.4	1.1 ± 0.2
	20 min	4.8 ± 0.8	1.6 ± 0.8
PAN-PCL	10 min	4.6 ± 0.1	1.4 ± 0.1
	20 min	4.1 ± 1.6	1.5 ± 0.9

**Table 2:** Value of the contact angle test on the uncoated and ZrO2_Ag_10- and ZrO2_Ag_20-coated membranes.

	Sample	Contact Angle (°)
PCL	Uncoated	107.9 ± 1.9
	ZrO2-Ag_10	97.4 ± 1.5
	ZrO2-Ag_20	127.5 ± 2.1
PAN-PCL	Uncoated	/
	ZrO2-Ag_10	98.4 ± 1.5
	ZrO2-Ag_20	88.9 ± 1.4

**Table 3:** Summary of the filtration results on PAN-PCL.

Bacterium	Sample	CFUs Before Filtration	CFUs After Filtration	Growth on Membrane
*B. subtilis*	Uncoated	88	1	Yes
	ZrO2_Ag_10	-	-	No
	ZrO2_Ag_20	86	10	No
*L. monocytogenes*	Uncoated	TM	TM	Yes
	ZrO2_Ag_10	27	14	No
	ZrO2_Ag_20	26	18	Non
*E. coli*	Uncoated	54	39	No
	ZrO2_Ag_10	46	44	No
	ZrO2_Ag_20	55	54	No

TM: Too Many to Count.

## Data Availability

The data presented in this study are available upon request from the corresponding author.
